# Virtual reality simulation improves performance metrics in total knee arthroplasty training: A single‐centre longitudinal study

**DOI:** 10.1002/jeo2.70538

**Published:** 2025-11-14

**Authors:** Vanessa Kley, Sabrina Chelli, George Avram, Felix Amsler, Alexandra Leica, Randa Elsheikh, Michael T. Hirschmann, Matthias Koch

**Affiliations:** ^1^ University Department of Orthopedic Surgery and Traumatology, Centre of Musculoskeletal Diseases, Kantonsspital Baselland Bruderholz Switzerland; ^2^ Department of Clinical Research, Research Group Michael T. Hirschmann, Regenerative Medicine & Biomechanics University of Basel Basel Switzerland; ^3^ Amsler Consulting Basel Switzerland; ^4^ Department of Trauma Surgery University Hospital Regensburg Regensburg Germany

**Keywords:** knee arthroplasty, simulation, surgical education, surgical training, virtual reality

## Abstract

**Purpose:**

Surgical training of orthopaedic residents is considered challenging. Limited knowledge is currently available regarding alternative approaches such as virtual reality (VR)‐based training for total knee arthroplasty (TKA). The aim of this study was to investigate the effect of VR‐based training on residents' surgical performance and the individual learning curves in VR‐simulated TKA.

**Methods:**

In an educational longitudinal study of learning performance across multiple time points, five sessions were completed on a VR TKA simulator by residents at weekly intervals. The sessions included a knowledge (multiple‐choice questions) and skills part, in which a VR‐simulated TKA was performed using a fully immersive commercially available VR simulator (Fundamental VR Surgery HapticVR™ Simulator; FundamentalSurgery, London, GB).

**Results:**

The results of 17 orthopaedic residents were analysed. Significant findings with strong effect sizes were observed for the primary endpoints in the surgical skill evaluation. A significantly higher number of the 16 surgical steps were successfully completed following simulator training (mean diff. ± SD: 0.11% ± 0.12%, *p* = 0.005; partial eta squared = 0.509; Cohen's *d* = 1.11) by simulator training. The time per step decreased (mean diff. ± SD: −22.73 ± 11.8 s; *p* = 0.000; partial eta squared = 0.807; Cohen's *d* = 1.63) and an improvement in surgical site gaze (SSG) (mean diff. ± SD: 9.65% ± 4.18%; *p* = 0.000; partial eta squared = 0.874; Cohen's *d* = 2.70) was observed.

**Conclusions:**

VR‐based training for TKA was found to have a significant positive effect on the learning curve. Improvements were observed in steps completed, time required and SSG. VR‐based training can therefore be considered as a safe and effective alternative in the training of orthopaedic residents.

**Level of Evidence:**

Level III.

**Abbreviations**
3D: three dimensional
CI: confidence interval
ESSKA: European Society for Sports Traumatology, Knee Surgery and Arthroscopy
MC: multiple choice
OR: operating room
PGY: post‐graduate year
SSG: surgical site gaze
TKA: total knee arthroplasty
UKA: unicompartmental knee arthroplasty
VR: virtual reality

## INTRODUCTION

Surgical skills training in orthopaedic surgery has become challenging in the last decade. Due to the advances in technology, increased attention to patient safety and ethics, growing bureaucracy, and need for greater cost efficiency in clinical practice, alternative methods of surgical training outside the operating room (OR) have been required [3, 12, 22]. In addition to supervised surgery on real patients, there are conventional training methods for surgeons, including learning from guidelines and videos or practicing on cadaveric simulations and anatomic models. But as beneficial as these methods are, they all have limitations regarding the practical relevance and daily availability [16, 21]. In contrast to that, one of the emerging technologies to acquire surgical skills is the use of virtual reality (VR) simulators. Fully immersive VR systems utilise headsets with attached displays to create an interactive virtual environment, while portable controllers provide kinesthetic haptic feedback, giving users the impression of being situated in a real OR setting [9]. The benefit of VR training is that orthopaedic procedures can be practiced in a risk‐free environment, accessible at any time and without the need for supervision by a senior physician. Direct feedback is generated based on performance data collected by the simulator, allowing repetitive training cycles and targeted, skill‐oriented practice to address individual weaknesses [9, 20, 21]. Numerous studies have examined the use of VR simulators in arthroscopy training, but only a few have investigated the use of VR simulators for open knee procedures [1, 6], which involve less standardised techniques and a broader range of complex tasks, making them more difficult to simulate [11]. However, today's technical advances in VR simulation make it possible to simulate open procedures such as total knee arthroplasty (TKA) [9]. TKA is one of the most efficacious and commonly performed orthopaedic surgeries [23]. Consequently, knee arthroplasty has become an important part of the core curriculum established by the European Society for Sports Traumatology, Knee Surgery and Arthroscopy (ESSKA) [15]. To perform TKA, it is necessary to master various surgical steps such as the femoral and tibial cuts, alignment and patellar resurfacing as well as correct tissue and instrument handling [8]. Previous studies have shown that it is essential for residents to gain experience to shorten operation time [5, 10] and reduce implant malalignment [13]. However, further research is needed to evaluate the effectiveness of VR for surgical training purposes. Analysing the learning curve is crucial in order to determine whether regular practice on the simulator, combined with multisensory real‐time feedback, leads to measurable improvements in surgical skills. This information can influence future strategies for the education and training of orthopaedic residents and will determine whether greater integration of VR technologies is warranted. The aim of the present study was to investigate the effect of VR‐based training on residents' surgical performance and individual learning curves in VR‐simulated TKA.

## METHODS

### Study population

This single‐centre study was conducted at the University Department of Orthopaedic Surgery and Traumatology at the Kantonsspital Baselland, Bruderholz, Switzerland. The study was approved by the local Ethics Committee. The research project was classified as a quality control project and therefore did not fall under the scope of the Human Research Act. Written consent was obtained from each participant. An educational longitudinal study design with multiple time points was used to analyse the effect of VR‐based training on the learning curve for performing a VR‐simulated TKA. Based on a prior case number planning (*N* = 16 participants required), a total of 17 orthopaedic residents were included. Participation was voluntary and without any financial or other compensation. A questionnaire (Appendix Questionnaire 1) was used to collect baseline characteristics such as age, post‐graduate year (PGY) or hand dominance for each resident. In addition, data about prior experience regarding TKA, anatomy or video games/VR were collected, and the participating residents judged their ability to perform TKA on a Likert scale (scale of 1 to 5, where 1 indicates no prior knowledge and 5 excellent knowledge).

### Training protocol

Weekly training or assessment sessions were performed by each participant over a 5‐week period. A total of three assessments and two training sessions were conducted, each lasting around 40 min. The sessions alternated between assessment and training, starting with an assessment session.

The sessions included two elements: a knowledge part and a skills part. The knowledge evaluation included multiple‐choice (MC) questions on preoperative management (preoperative test), procedural knowledge (intraoperative test), and postoperative management (postoperative test). The respective tests (preoperative, intraoperative, and postoperative) were compared within their respective categories. The skills evaluation required the performance of a virtual TKA using the VR simulator.

**Figure 1:**
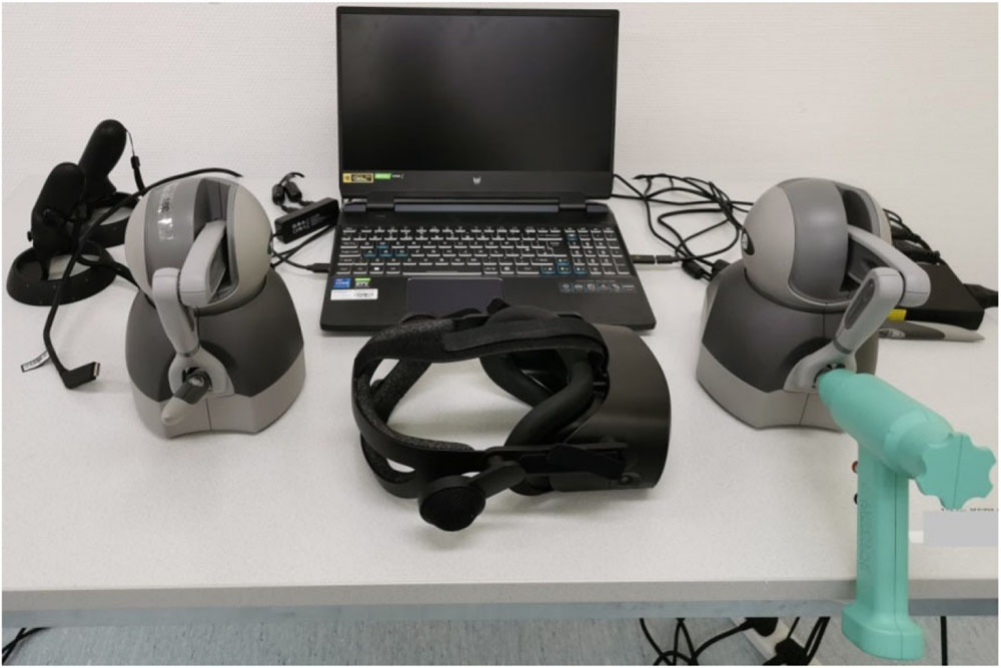
Fully immersive, established virtual reality simulator with head‐mounted display and haptic handheld controllers including three‐dimensional printed drill and saw.

To create a realistic OR simulation, the VR simulator was equipped with a head‐mounted display that allowed the operator to visualise the OR and a patient's knee (Figure 1). X‐rays (XR) were shown on a board for the surgeon to interpret. The same set of XR images was used for every TKA session to ensure standardisation. Additionally, there were two kinesthetic haptic arm devices providing the surgeon with a realistic tactile feedback during drilling or sawing. The standard pointers attached to the haptic arms were replaced with 3D‐printed replicas of a surgical drill and a saw (Figure 1). To mimic a more realistic OR experience, the system setup, such as table height or position of the haptic arms, was adjusted individually for each participant before training and testing.

The first session started with a standardised introduction and a brief demonstration of the simulator's handling. In order to familiarise the participants with the VR simulator and the handheld haptic devices, an instructional session was held and the programme's tutorial was executed in a standardised manner.

**Figure 2:**
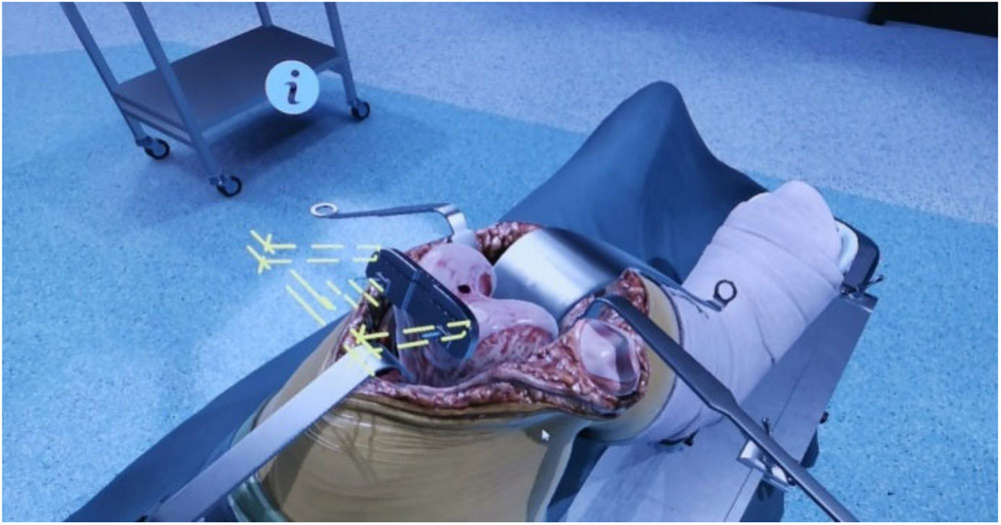
Activated guidance lines of the simulator's visual help (yellow lines).

A baseline test was conducted to measure the initial skills and establish a baseline for the subsequent analysis of performance and learning curve. During each session, the participants performed the same standardised workflow of a virtual TKA. There was no variation in terms of alignment. For each surgical step, written instructions were shown on the screen. The first, third and fifth sessions were designated as test sessions, whereas the sessions occurring between these sessions were training sessions. During the training sessions, visual, audio, and text guidance were available and could be activated or deactivated by participants at any step. For example, when visual guidance was activated, guidance lines were projected onto the patient's knee (Figure 2). The test sessions allowed no visual, audio or text guidance. Regarding the knowledge part, there was a difference between the test and training sessions, too. All sessions included the intraoperative test with six MC questions during the TKA procedure. The preoperative and postoperative tests, consisting of ten and nine MC questions (Appendix Questions of the pre‐/intra‐/post‐operative test), respectively, were added to the two test sessions. After the final session, the participants were invited to complete a brief questionnaire (Appendix Questionnaire 2) designed to evaluate their experience with the simulator. Detailed information concerning the schedule of assessments is specified in the appendix.

### Primary and secondary endpoints

The surgeons’ skills were analysed by the VR simulator based on their performance during each step (16 in total) of the TKA procedure. Specific measurements were tracked simultaneously, including economy of movement, 3D spatial awareness, submillimeter accuracy, surgical site gaze (SSG; percentage of time spent looking at the surgical site), and attention for the surrounding soft tissues during surgery. Following each simulator session, the total procedure duration (minutes), the SSG (%), and a pass or fail grade for each step performed were provided to the participant.

The primary endpoint of the study was determined as the number of steps passed (X/16). The percentage of steps passed was referred to as the ‘result’ and displayed as a percentage on the learning curve. Secondary endpoints were defined as the improvement in the total duration (min) as well as the improvement in the overall SSG (%). In addition, changes in procedural knowledge were analysed using the MC questions. All scores and parameters were generated by the simulator and made available by Fundamental Surgery, London, UK.

### Statistical analysis

Performance data were generated by the VR simulator and statistically analysed. Learning curves were created based on the data for the result, SSG and time. To describe the learning curves, repeated‐measures analysis of variance was calculated for time points t1, t3 and t5. Correlations were performed using Pearson correlation coefficients. In addition, the learning curves of the individual participants were presented graphically. A one‐sided significance level *p* < 0.05 was used for all tests. Statistical analysis was conducted using SPSS software, version 26.0 (SPSS, Chicago, IL, USA). The required number of study participants was previously determined through case size calculation, which indicated that a minimum of 16 participants had to be included. For the paired comparison between t1 and t5 with a one‐sided *p* < 0.05, an effect size *d* = 0.43 could be achieved with a statistical power of 80%. Since strong learning effects were expected, an effect size of *d* = 0.43 was considered sufficiently low for this study.

### Study aim and hypothesis

The primary objective of this study was to generate and analyse individual learning curves for knee arthroplasty in orthopaedic residents using an accredited orthopaedic surgery simulator. The simulator used was a fully immersive VR system providing a kinesthetic haptic feedback.

The study was based on the hypothesis that a measurable learning curve could be observed in orthopaedic residents practicing with the VR simulator. It was expected that the participating residents would demonstrate improvements in their surgical skills when performing a virtual TKA, reflected by an increase in the steps completed, time efficiency, and surgical site gaze (SSG).

## RESULTS

The present study includes orthopaedic residents from all training levels. The mean orthopaedic training year was calculated to be 3.8 years. Complete participant characteristics are shown in Table 1.

**Table 1:** General characteristics of study participants.

Characteristic		Total (*n* = 17)
Demographics		
Age	Mean (SD) (years)	30.9 (3.6)
Sex	*n* (%)	
Male		76.5
Female		23.5
Training year	Mean (SD) (years)	
total		4.4 (2.3)
orthopaedic		3.8 (2.2)
Training year (total)	*n* (%)	
PGY‐1		5.9
PGY‐2		17.6
PGY‐3		17.6
PGY‐4		5.9
PGY‐5		23.5
PGY‐6		17.6
PGY‐7		5.9
PGY‐10		5.9
Training year (orthopaedic)	*n* (%)	
PGY‐1		17.6
PGY‐2		17.6
PGY‐3		11.8
PGY‐4		5.9
PGY‐5		23.5
PGY‐6		17.6
PGY‐8		5.9

Abbreviation: PGY, post‐graduate year.

All participating residents had at least observed or assisted in TKA procedures before. The practical experience as a primary surgeon was reported as minimal or absent (88.3%). The majority of study participants (82.3%) rated their knowledge of knee anatomy as moderate to high, while 76.5% self‐assessed their prior knowledge of TKA as little or moderate. However, some degree of prior knowledge regarding TKA was reported by all participants. With the exception of one individual, all participants reported having played video games, though for the majority, this engagement was infrequent. Four subjects (23.5%) reported great to extensive experience with playing video games. Almost all participants (94.1%) reported little to no experience with VR or immersive games. Two participants indicated that they had never used orthopaedic simulators before. Detailed information on participants' previous experience is presented in Table 2.

**Table 2:** Experience/knowledge level of study participants (*N* = 17).

Experience/knowledge					
	TKA cases			Knowledge	
	Observed, %	Assisted, %	Performed (as primary surgeon), %	Anatomical structures of the knee, %	TKA, %
No	0.0	5.9	47.1	0.0	5.9
Little	17.6	11.8	41.2	11.8	35.3
Moderate	23.5	23.5	5.9	58.8	41.2
Great	41.2	47.1	0.0	23.5	11.8
Extensive	17.6	11.8	5.9	5.9	5.9

	Use/experience of				
	Drill/saw in OR, %	Domestic drill/saw, %	Video games, %	VR/immersive games, %	Orthopaedic simulations, %
No	5.9	0.0	5.9	58.8	11.8
Little	52.9	41.2	35.3	35.3	64.7
Moderate	23.5	23.5	35.3	0.0	17.6
Great	11.8	29.4	17.6	0.0	0.0
Extensive	5.9	5.9	5.9	5.9	5.9

Abbreviations: TKA, total knee arthroplasty. VR, virtual reality.

**Figure 3:**
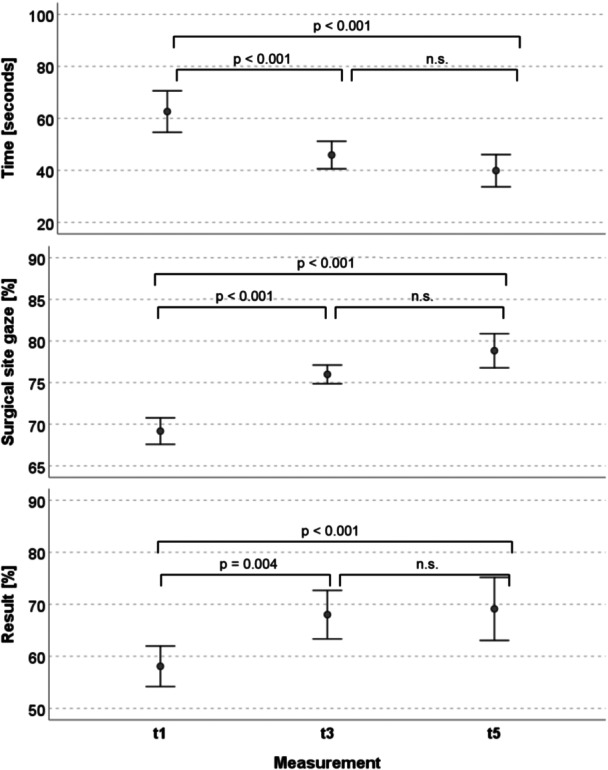
Mean time needed in seconds and surgical site gaze and result in percentage. Progression from assessment session 1 (t1) to session 3 (t3) to session 5 (t5). The circle shows the mean values and the error bars show mean and 95% confidence interval. Probability *p* for one‐sided paired *t*‐test.

A significant improvement was demonstrated for both the primary and secondary endpoints. A significantly higher number of the 16 surgical steps assessed were passed after training with the VR simulator compared to the baseline test (t 1: 58% to t 5: 69%, *p* = 0.001; Cohen's *d* = 1.11, *p* = 0.001) (Table 3). This improvement was also reflected in the overall results (Figure 3). An extraordinary increase was particularly observed between t1 and t3. A significant reduction with a strong effect size (Cohen's d 1.63, *p* = 0.000) was detected in the time required to complete the 16 surgical steps from session one (t1) to session three (t3) and to session five (t5) (Figure 3). As shown in Table 3, the time per step decreased significantly from 62.6 s to 39.8 s (*p* < 0.001). According to Figure 3, a significant improvement in SSG was observed, increasing from 69.2% to 78.8% (*p* < 0.001). This difference between the VR sessions was also significant and demonstrated a strong effect size (Cohen's d 2.70, *p* = 0.000) (Table 3). Regarding the correlations for these three variables between t1 and t5, significant results were found only for the time needed (Table 3).

**Table 3:** Detailed results of the simulator‐generated data.

Variable	Mean ± SD T1	Mean ± SD T5	Partial eta squared	*p* value	Pearson r	*p* value
**Result**	0.58 ± 0.08	0.69 ± 0.12	0.509	0.005	0.29	0.128
**Time**	62.6 ± 15.51	39.87 ± 12.09	0.807	0.000	0.66	0.002
**SSG**	69.18 ± 3.09	78.83 ± 3.99	0.874	0.000	0.33	0.101
**Pre‐result**	0.64 ± 0.17	0.88 ± 0.13	0.748	0.000	0.58	0.008
**Intra‐result**	0.82 ± 0.15	0.95 ± 0.13	0.828	0.000	0.24	0.173
**Post‐result**	0.63 ± 0.16	0.88 ± 0.09	0.834	0.000	0.74	0.001
**Pre‐time**	210.06 ± 61.71	124.76 ± 33.41	0.710	0.000	0.35	0.084
**Intra‐time**	86.71 ± 26.13	44 ± 12.45	0.839	0.000	0.40	0.058
**Post‐time**	0.63 ± 0.16	0.88 ± 0.09	0.621	0.001	0.74	0.001

Abbreviations: SD, standard deviation; Pre, preoperative test; Intra, intraoperative test; Post, postoperative test.

The examination of procedural knowledge based on the MC questionnaires in the pre‐, intra‐ and postoperative tests showed significant results with strong effect sizes (Table 3). Significant improvements were observed in both time and result. From t1 to t5, the questions were answered 85.3 s faster in the preoperative test, 42.7 s faster in the intraoperative test, and 50.9 s faster in the postoperative test (*p* < 0.001). Significant improvements were also observed in the results. In the preoperative test at t5, a score of 88% was achieved compared to 64% at t1 (*p* < 0.001). In the intraoperative test, scores improved from 82% (t1) to 95% (t5) (*p* < 0.005), while in the postoperative test, scores increased from 63% (t1) to 88% (t5) (*p* < 0.001). Thus, the overall pass mark of 80% was reached on average at t5. With the exception of two values, the correlations between t1 and t5 were low and not significant (Table 3).

Evaluating the effect of experience, no significant correlation was found between the residents’ experience levels and their performance in the baseline test or their learning curve. In contrast, experience was found to correlate more strongly with the skills assessment than with the values measured in the simulation. In particular, the ‘Ability to perform a TKA’ t1 and t5 was strongly correlated with the ‘TKA cases performed (as primary surgeon)’ (t1: Pearson 0.88; *p* < 0.001; t5: Pearson 0.84; *p* < 0.001) and the ‘Knowledge level regarding TKA’ (t1: Pearson 0.8; *p* < 0.001; t5: Pearson 0.76; *p* < 0.001). In addition, highly significant correlations were observed between the ‘Ability to perform a TKA t1’ and ‘Use of drill/saw in OR’ (Pearson 0.72; *p* < 0.001), ‘Experience of VR/immersive games’ (Pearson 0.72; *p* < 0.001) and ‘Use of orthopaedic simulations’ (Pearson 0.74; *p* < 0.001).

Investigating the participants’ opinion on the realism of the virtual TKA, the majority of respondents generally rated the simulation as realistic. More than half of the participants reported that the simulation looked (58.8% agreement) and sounded (52.9% agreement) realistic. The clinical scenario was also considered realistic (64.7% agreement). Opinions differed, however, regarding whether the simulation felt realistic (Table 4).

**Table 4:** Participant feedback on the VR simulator.

	Simulation			
	Looked realistic, %	Felt realistic, %	Sounded realistic, %	Realistic clinical scenario, %
Strongly disagree	5.9	17.6	0.0	0.0
Disagree	11.8	29.4	17.6	17.6
Neither agree or disagree	23.5	11.8	23.5	17.6
Agree	58.8	41.2	52.9	64.7
Strongly agree	0.0	0.0	5.9	0.0

Abbreviation: VR, virtual reality.

**Figure 4:**
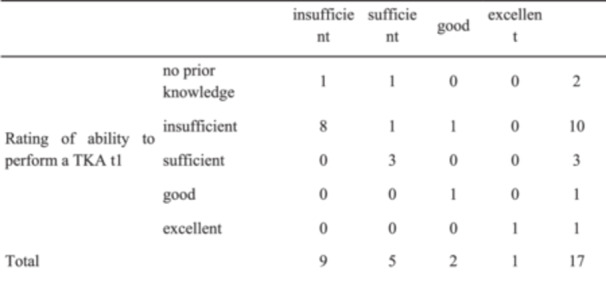
Rating of self‐assessment regarding the ability to perform a total knee arthroplasty (TKA) at the timepoints t1 and t5. The fields marked in green show that these participants improved in their ability to perform a TKA between sessions 1 and 5 according to their self‐assessment. It remained the same for the remaining participants. Rating of ability to perform a TKA t5 Crosstabulation.

With regard to the learning effect, it was reported that the VR simulation primarily improved theoretical knowledge (64.7% agreement, 5.9% strong agreement) and understanding of the different surgical steps (58.8% agreement, 29.4% strong agreement). For detailed information, see Appendix (Table 5). In total, an improvement based on self‐assessment was reported by four participants (23.5%) as a result of the VR training (Figure 4).

**Table 5:** Participant feedback on the learning effect of the VR simulation.

	Simulation improved/trained			
	Theoretical knowledge, %	Technical skills, %	How to use instruments, %	Different surgical steps, %
Strongly disagree	11.8	11.8	5.9	0.0
Disagree	5.9	17.6	47.1	5.9
Neither agree or disagree	11.8	41.2	11.8	5.9
Agree	64.7	23.5	29.4	58.8
Strongly agree	5.9	5.9	5.9	29.4

Abbreviation: VR, virtual reality.

## DISCUSSION

The most important finding of the present study was the significant effect of the VR‐based TKA training on the learning curve of orthopaedic residents. Indeed, the current literature describes various approaches and aspects using VR to train orthopaedic surgeons outside of the OR, as education has become increasingly challenging in recent years [3, 12, 19, 22]. However, most research has focused mainly on the use of VR simulators for arthroscopy training, with limited investigation into open surgical procedures such as TKA [2, 4, 11, 14, 18, 24, 25, 26]. Consequently, current literature lacks data on detailed information about the learning progress of orthopaedic residents using immersive VR for TKA training.

The present study shows for the first time that results and SSG significantly improved, and time needed significantly decreased when performing a TKA using and training with an immersive VR simulator. These findings are in accordance with similar studies demonstrating that immersive VR simulators are an effective training method for open surgical procedures, improving accuracy, reducing procedural time, and enhancing skill acquisition [20]. The aim of this study was to determine more precisely the effect of VR‐based training in orthopaedic surgery. Therefore, individual learning curves for TKA in orthopaedic residents, generated using an accredited orthopaedic surgery simulator, were analysed. The results demonstrated the existence of a clear learning curve for TKA through VR simulator training.

Significant improvement was observed in all three evaluated categories: result, SSG, and time. The overall outcome of the surgical steps passed (result) improved by a mean of 11% (*p* = 0.001), while the time required decreased by a mean of 22.7 s (*p* < 0.001) and the SSG increased by a mean of 9.7% (*p* < 0.001) from the baseline to the final test. According to Cohen's guidelines, effect sizes can be interpreted as small (*d* = 0.01), medium (*d* = 0.06) or strong (*d* = 0.14). These findings confirm previous studies showing the beneficial learning effect of VR training compared to conventional training methods in orthopaedic surgery [3, 9, 20]. Several studies have specifically demonstrated this effect for arthroscopy training [2, 5, 25, 26]. Recent literature has also described an advantage of VR training compared to conventional preparation methods for arthroplasty [11, 16, 17]. McKinney et al. [21] demonstrated that residents performed a higher number of correct and faster surgical steps during unicompartmental knee arthroplasty (UKA) following VR training compared to preparation using technical guides. Similarly, Zaid et al. [27] reported that VR training was not inferior to guide preparation in terms of procedure time and Objective Structured Assessment of Technical Skills (OSATS) score for UKA. Additionally, the present study showed that VR training not only leads to an improvement in surgical skills, but also to an increase in theoretical knowledge. The percentage of correctly answered MC questions increased significantly from the first to the last simulator session. The participants improved by 24% (*p* < 0.001) on the preoperative test, by 13% (*p* = 0.004) on the intraoperative test and by 24% (*p* < 0.001) on the postoperative test. Similar results were described by Lohre et al. [17], who observed higher knowledge scores for simulated arthroplasty in expert groups compared to residents. In contrast, no correlation between the participants’ experience level and performance in VR simulation was identified in the present study. This finding contrasts with previous literature, which reported a positive correlation between participant experience and performance in virtual arthroplasty [7, 17]. This lack of correlation might be explained by the composition of the evaluated group. Unlike the existing literature that compares completely distinct groups such as residents, scrub nurses, or expert/consultant surgeons, the present study included only residents from different postgraduate years (PGYs), resulting in more homogeneous experience levels. This finding is particularly surprising, as the majority of residents perceived the simulation as realistic; however, it might be explained by the wide range of practical surgical experience of the individual residents.

The immersive effect of the VR simulator and the subjective learning effect were also evaluated. Most participants agreed that the simulation looked (58.8%) and sounded (52.9%) realistic and is able to imitate a realistic clinical scenario. Nevertheless, a weakness in the respondents’ view was the haptic feedback of the system, which has also been reported by Lohre et al [17]. In the current literature, participants were generally supportive of the use of VR simulators in surgical training [7, 11, 17, 27]. Additionally, participants reported increased confidence in performing the trained procedures in previous research, as also described by four residents in the present study [27]. Furthermore, the simulation was reported to improve both technical knowledge and the performance of surgical steps. Similar findings were also observed by Lohre et al [17]. Participants in this study also found VR training helpful in teaching technical skills (glenoid preparation and exposure, retractor placement) performed during surgery.

Despite its strengths and unique data collection, several limitations of the present study should be considered when interpreting the results. Within this longitudinal study, the individual learning effect of VR‐based arthroplasty training was analysed and inter‐individually compared to the baseline level without a control group or alternative training method. Thus, no statement can be made regarding the effectiveness of VR‐based arthroplasty training compared to conventional educational methods for orthopaedic surgeons, such as technical guides or cadaveric simulations. Furthermore, no comparison was made with TKA procedures performed under real OR conditions. Although a priori case number planning was conducted, the sample size of 17 participants from a single centre was small, resulting in only a few residents per PGY category. This might be one reason for the lack of correlation between the different experience levels and the performance in the simulation. Nevertheless, the required number of participants determined by the power analysis was achieved to generate statistically significant results. It is challenging to recruit large numbers of residents in a single centre. Additionally, simulator‐generated data without any verification of the learning effect observed in real TKA was analysed. Therefore, no statement can be made about the participants’ real‐life surgical performance. Although there is evidence in the literature that VR training improves cadaveric joint replacement, it is not clear whether it improves the ability to perform a real TKA. Further research is needed to assess the transferability of VR training to real life.

## CONCLUSION

The present study demonstrated that orthopaedic residents show a relevant learning curve in performing a TKA on the VR simulator for TKA. They improved in the number of surgical steps completed, time required and SSG after a total of 5 weekly sessions. Therefore, VR simulator training could be considered as a possible safe alternative in the preparation and training of residents.

## AUTHOR CONTRIBUTIONS

All authors have contributed to the conception and design of this study, acquisition of data, drafting the article, its revision, and all authors approved the final draft of the submitted article. All authors have read and agreed to the published version of the manuscript.

## CONFLICT OF INTEREST STATEMENT

The authors declare no conflicts of interest.

## ETHICS STATEMENT

According to the Ethics Committee Northwest and Central Switzerland (EKNZ), this research project does not fall under the scope of the Human Research Act, because the project is classified as a quality control project. An authorisation from the ethics committee was therefore not required and the EKNZ is not responsible for its review.

## Supporting information

Supporting information.

## Data Availability

The datasets used and/or analysed during the current study are available from the corresponding author on reasonable request.
