# Supplementary material for: Virtual reality simulation improves performance metrics in total knee arthroplasty training: A single‐centre longitudinal study
Source: J Exp Orthop. 2025 Nov 14;12(4):e70538. doi: 10.1002/jeo2.70538 (PMC12616389; doi:10.1002/jeo2.70538)
Supplement: Supplementary file 1 — Supporting information. [file JEO2-12-e70538-s001.docx]

## APPENDIX

##

## Flowchart of the appointments per participant

| **Study Appointment** | **Before the 1^st^ appointment** | **1** | **2** | **3** | **4** | **5** |
| --- | --- | --- | --- | --- | --- | --- |
| **Date** | Week -1 | Week 0 | Week 1 | Week 2 | Week 3 | Week 4 |
| **Duration** | 5 min | 40-50 min | Max.  40 min | Max.  40 min | Max.  40 min | 40-50 min |
| **Questionnaire 1** | ✓ |  |  |  |  |  |
| **Tutorial** |  | ✓ |  |  |  |  |
| **Knowledge test**  **(MC questions)** |  | ✓ |  | ✓ |  | ✓ |
| **Perform a guided virtual training TKA** |  |  | ✓ |  | ✓ |  |
| **Perform a unguided virtual assessment TKA** |  | ✓ |  | ✓ |  | ✓ |
| **Questionnaire 2** |  |  |  |  |  | ✓ |

## Questionnaire 1

| Name | ____________________________________ | | | | |
| --- | --- | --- | --- | --- | --- |
| Age  Resident year   1. In total 2. As orthopedic resident | ____________________________________  ____________________________________  ____________________________________ | | | | |
| Hand dominance | ____________________________________ | | | | |
|  |  |  |  |  |  |
|  | 1  no prior experience/ knowledge | 2  little  experience  /knowledge | 3  moderate  experience/  knowledge | 4  great  experience/  knowledge | 5  extensive prior experience/  knowledge |
| TKA cases observed | ○ | ○ | ○ | ○ | ○ |
| TKA cases assisted | ○ | ○ | ○ | ○ | ○ |
| TKA cases performed (as primary surgeon) | ○ | ○ | ○ | ○ | ○ |
| Use of domestic drill/saw | ○ | ○ | ○ | ○ | ○ |
| Use of drill/saw in operating room | ○ | ○ | ○ | ○ | ○ |
| Knowledge of anatomical structures of the knee | ○ | ○ | ○ | ○ | ○ |
| Knowledge level regarding TKA | ○ | ○ | ○ | ○ | ○ |
| Use of video games | ○ | ○ | ○ | ○ | ○ |
| Experience of VR/Immersive games | ○ | ○ | ○ | ○ | ○ |
| Use of orthopedic simulations | ○ | ○ | ○ | ○ | ○ |
|  | 1 | 2 | 3 | 4 | 5 |
|  | no prior knowledge | insufficient | sufficient | good | excellent |
| How do you rate your ability to perform a TKA? | ○ | ○ | ○ | ○ | ○ |

###

## Questionnaire 2

| Name | ____________________________________ | | | | | | |
| --- | --- | --- | --- | --- | --- | --- | --- |
|  | 1 | | 2 | | | 3 | |
|  | too easy | | just right | | | too hard | |
| What was the level of the MC-questions? | ○ | | ○ | | | ○ | |
| What was the level of technical implementation of the TKA? | ○ | | ○ | | | ○ | |
|  | 1 | | 2 | | | 3 | |
|  | not enough | | just enough | | | too much | |
| How did you find the number of sessions in total? | ○ | | ○ | | | ○ | |
| How did you find the number of training sessions? | ○ | | ○ | | | ○ | |
|  | 1 | 2 | | 3 | 4 | | 5 |
|  | Strongly disagree | Disagree | | Neither agree nor disagree | Agree | | Strongly agree |
| Simulation looked realistic | ○ | ○ | | ○ | ○ | | ○ |
| Simulation felt realistic | ○ | ○ | | ○ | ○ | | ○ |
| Simulation sounded realistic | ○ | ○ | | ○ | ○ | | ○ |
| Realistic clinical scenario | ○ | ○ | | ○ | ○ | | ○ |
| Simulation improved theoretical knowledge | ○ | ○ | | ○ | ○ | | ○ |
| Simulation improved technical skills | ○ | ○ | | ○ | ○ | | ○ |
| Simulation trained how to use instruments | ○ | ○ | | ○ | ○ | | ○ |
| Simulation trained the different surgical steps | ○ | ○ | | ○ | ○ | | ○ |
| If you disagree with any of the statements above, why? | | | |  |  | |  |
|  | 1 | 2 | | 3 | 4 | | 5 |
|  | no prior knowledge | insufficient | | sufficient | good | | Excellent |
| How do you rate your ability to perform a TKA? | ○ | ○ | | ○ | ○ | | ○ |

## Questions of pre-operative test

**MC-Question 1**

Which of these is considered a contraindication for a primary total knee replacement?

A Extensor mechanism dysfunction

B Previous knee surgery

C Amputation of the contralateral leg

D A BMI of 35 and above

**MC-Question 2**

Which condition is a contraindication for total knee replacement?

A Osteoarthritis

B Knee arthrodesis

C Inflammatory arthritis

D Osteonecrosis

**MC-Question 3**

Which ligament in total knee replacement can be retained or removed based on the implant

type?

A Anterior cruciate ligament

B Lateral collateral ligament

C Medial collateral ligament

D Posterior cruciate ligament

**MC-Question 4**

Which approach for total knee replacement could be extended?

A Subvastus approach

B Medial parapatellar approach

C Lateral parapatellar approach

D Midvastus approach

**MC-Question 5**

What is templating?

A The process of trialing implants during the surgery

B The process of ensuring implant compatibility

C The process of pre-operatively anticipating the size and position of implants

D The process of planning the approach and the flow of the surgery

**MC-Question 6**

What is the first step in the process of templating?

A Obtaining appropriate radiographs with a calibration tool

B Recording patient measurement information

C Establishing limb length discrepancy

D Reviewing relevant patient history

**MC-Question 7**

Which X-ray view is at least valuable in ascertaining the size and position of total knee

replacement implants?

A Rosenberg view

B Antero-posterior

C Patellofemoral

D Lateral view

**MC-Question 8**

What instability is avoided by reproducing the tibial slope in a total knee replacement?

A Instability in extension

B Varus instability

C Instability in flexion

D Valgus instability

**MC-Question 9**

In a standard total knee replacement what is the angle of the proximal tibial cut?

A 6 degrees of valgus

B 6 degrees of varus

C 3 degrees of valgus

D 0 degrees (neutral)

**MC-Question 10**

What is the intramedullary femoral guide used to estimate in total knee replacement?

A The difference between the mechanical and anatomic axis of the femur

B The anatomic axis of the femur

C The rotation of the femoral implant

D The center of the hip

## Questions of intra-operative test

**MC-Question 1**

Looking at the patient, which structure is the MCL?

A

B

C

D

**MC-Question 2**

Looking at the patient, which structure is the LCL?

A

B

C

D

**MC-Question 3**

Why is the amount of resection of the distal femur important?

A It affects the extension gap

B It affects the femoral implant size

C It affects the tibial implant size

D It affects the flexion gap

**MC-Question 4**

What does the proximal tibial resection impact the most?

A The tibial baseplate rotation

B The tibial implant size

C The femoral implant size

D The flexion and extension gaps

**MC-Question 5**

Which artery is at risk when performing the proximal tibial resection?

A Radial artery

B Posterior tibial artery

C Popliteal artery

D Femoral artery

**MC-Question 6**

Which nerve is at risk when performing proximal tibial resection?

A Tibial nerve

B Sciatic nerve

C Superficial fibular nerve

D Sural nerve

## Questions of post-operative test

**MC-Question 1**

Which of these are valid DVT prophylaxis measures following total knee replacement?

A Early mobilization

B Blood-thinning medication

C Adequate hydration

D All of the above

**MC-Question 2**

When should the tourniquet be deflated?

A Before the final implant checks

B Immediately after the closure of the arthrotomy

C Before the closure

D After placement of the surgical dressings

**MC-Question 3**

At the end of the procedure which arterial pulses should be assessed and compared?

A Femoral

B Popliteal

C Posterior tibial

D Radial

**MC-Question 4**

Which X-ray views are the minimum necessary for acceptable postoperative evaluation?

A Oblique and patellofemoral

B Antero-posterior and patellofemoral

C Antero-posterior and lateral

D Lateral and Rosenberg

**MC-Question 5**

On which X-ray can the overhang of the components be most clearly visualized?

A Skyline

B Rosenberg

C Lateral

D Antero-posterior

**MC-Question 6**

On which X-ray can the tibial slope be evaluated?

A Lateral view

B Rosenberg view

C Skyline view

D Antero-posterior view

**MC-Question 7**

Which is the most common reason for total knee replacement failure?

A Ligament / flexion instability

B Aseptic component loosening

C Septic failure

D Periprosthetic fracture

**MC-Question 8**

What is the goal of a revision for a total knee replacement?

A Restoration of joint line

B Stable revision implants

C Extraction of components with minimal bone loss and destruction

D All of the above

**MC-Question 9**

Which complications must be evaluated on both AP and lateral post-operative X-rays?

A Fractures

B Patella loosening

C Excessive tibial slope

D Component rotation
